# The Effects of Physical Exercise on Saliva Composition: A Comprehensive Review

**DOI:** 10.3390/dj10010007

**Published:** 2022-01-05

**Authors:** Panagiotis Ntovas, Nikolaos Loumprinis, Panagiotis Maniatakos, Loukia Margaritidi, Christos Rahiotis

**Affiliations:** 1 Department of Operative Dentistry, University of Athens, 11527 Athens, Greece; pan.ntovas@gmail.com (P.N.); nikosloubr@hotmail.com (N.L.); 2 Private Practice, 11527 Athens, Greece; maniatakospanagiotis@gmail.com (P.M.); Loukm@gmail.com (L.M.)

**Keywords:** oral health, saliva, sports dentistry, physical exercise, sports medicine

## Abstract

Saliva consists of organic and inorganic constituents. During exercise, analysis of the saliva can provide valuable information regarding training stress, adaptation and exercise performance. The objective of the present article was to review the effect of physical exercise on saliva composition. The shift in the composition of the saliva, during and after a workout, reflects the benefits of exercise, its potential risks and the capability of the saliva to serve as a health indicator. The type and the frequency of training, the physical condition and the athletes’ general health influence the hormones, immunoglobulins and saliva enzymes. The correlation between saliva and physical exercise has to be further investigated and the available knowledge to be applied for the benefit of the athletes during sports activities.

## 1. Introduction

Salivary glands are exocrine organs that produce a large amount of fluid. Through the saliva, electrolytes and other substances are transferred from the inner of the glands to the oral cavity. The mean volume of the fluid is estimated to be 750 mL/day, which almost represents 20% of the overall plasma volume [1,2]. Apart from the major salivary glands (2–5 mL/min), saliva is also secreted by several minor glands at a rate of 0.5 mL/min [3].

Salivary glands are stimulated by parasympathetic cholinergic nerves and sympathetic adrenergic nerves [4]. Parasympathetic stimulation increases regional blood flow and saliva, consisting of low organic and inorganic components [5]. Sympathetic stimulation results in the saliva of low volume, containing high levels of K^+^ and proteins [6]. The autonomous nervous system regulates salivary secretion. Catecholamines might also play a role in the secretion of electrolytes and proteins [7]. During exercise, hormone response analysis can provide valuable information regarding training stress, adaptation, dehydration and exercise performance [8].

Saliva comprises 99% water and 1% organic and inorganic constituents [9,10]. Although saliva’s organic and inorganic components are usually present in low concertation, compared with the serum, some proteins such as a-amylase are synthesized in the glands and presented in higher levels [11,12,13]. Other organic components, which can be detected in the saliva are vitamin C, maltase, urea, uric acid, albumin, mucin, creatinine, amino acids, lactase and hormones such as testosterone, cortisol, etc. Moreover, amounts of CO_2_ are presented and so are immunoglobulins such as IgA, IgG, IgM [14].

The objective of the present article was to review the effect of physical exercise on saliva composition based on clinical studies.

## 2. Materials and Methods

The review focuses on clinical trials, directly investigating the effects of physical exercise or various sports on the composition of the saliva. A search was conducted in the PubMed and Scopus databases, implementing a Boolean strategy, to identify eligible articles. A literature search until September 2021 was performed, for articles written in English, without any restriction upon publication date. The search strategy included the following terms: (“physical exercise” OR “sports” OR “athletes”) AND (“saliva” or “salivary”). The references of the selected articles were also evaluated to identify themes that may fail to be detected by the search mentioned above strategy. 

## 3. Results

The search strategy revealed 4487 articles. Following the removal of duplicates, 2498 articles were remained. Based on the title and the abstract of the studies, 2.289 articles were removed. After the evaluation of the remaining articles against the eligibility criteria, a total of 125 articles were concluded in the present review. Physical exercise has a significant impact on the composition of saliva. Therefore, the data which were extracted from the selected studies are presented individually for each substance of the saliva, in the following categories: salivary secretion, lysozyme, lactoferrin, lactate, oral peroxides, nitric oxide, salivary A-amylase (sAA), salivary cortisol (S-Cortisol), steroids-testosterone, salivary immunoglobulin A (IgA), immunoglobulin G (IgG), immunoglobulin M (IgM), insulin-like growth factor 1 (s-IGF-1), salivary MicroRNAs and melatonin. In Table 1, the available studies for each of the components of the saliva, along with their primary outcomes are presented.

### 3.1. Salivary Secretion

Parasympathetic and sympathetic neural systems regulate saliva secretion. Each one has a different effect on its secretion. When the sympathetic neural system stimulates saliva secretion, it consists more of proteins such as α-amylase and cystatin. When the parasympathetic system stimulates saliva secretion, its volume is mainly increased [80]. Physical exercise seems to increase the salivary flow rate and protein (e.g., amylase, lysozyme and MUC5B) secretion [81]. The decreased saliva flow may influence the concentration of the saliva substances, such as the metabolites [55]. S-IgA’s protective role is dependent on its secretion rate [54].

It has been reported that in healthy individuals, unstimulated saliva is secreted at rest at the rate of 0.30–0.65 mL/min, whereas stimulated saliva flows at a rate of 1.5–6.0 mL/min [82]. Saliva flow rate increases during exercise to a secretion rate of 0.78–0.94 and decreases after recovery [73,74,75]. Under the physical exercise, as the flow rate of the saliva increases, the concertation of Na^+^ and HCO_3_ is raised. Na^+^, Ca^2+^, Cl^−^, HCO_3_ and proteins increase, whereas K^+^ shows little change [82]. Following the physical activity, the increase in salivary proteins may be associated with adrenergic activity [83]. Increased plasma catecholamines may also cause an a-amylase increase during exercise [84]. Salivary and serum cortisol increase linearly with the intensity of exercise [85]. Secretion of S-type cystatins and cystatin C is also increased by physical exercise [39].

Exercise, performed in normoxia and hypoxia, did not affect saliva flow rate or a-amylase concentrations. On the other hand, acute hypoxia increases mean saliva flow rate, both at rest and after exercise and a decrease in mean saliva K+ concentration, at rest and after exercise [39]. In addition, food consumption during the exercise increases saliva’s flow rate and the secretion of specific proteins, as lysozyme and α-amylase, but not s-IgA secretion [76].

### 3.2. Lysozyme and Lactoferrin 

Lysozyme and lactoferrin are the main saliva’s antimicrobial proteins. Lysozyme and lactoferrin act synergistically to augment immunity [45]. Lysozyme breaks down the polysaccharide wall of their cell, thus facilitating the destruction of mainly gram-positive bacteria [86]. Furthermore, exercise activates neutrophils, potentially causing the release of lysozyme and lactoferrin into the saliva [87,88]. The lysozyme concentration in the saliva and its secretion rate is negatively influenced by psychological stress [89]. Lactoferrin has anti-inflammatory and anti-microbial roles, preventing bacterial growth by sequestering ferric iron from the bacteria and directly interacting and damaging bacterial membranes [90,91].

Lower salivary lactoferrin concentrations were found in elite rowers than in the non-exercising control group over a training season [66]. Moreover, lactoferrin concentration in saliva decreased over a competitive training season in basketball players [29]. On the other hand, acute running increases lactoferrin and lysozyme expression in both men and women [27,92]. 

### 3.3. Lactate

Lactate is an essential source of energy for the metabolism of skeletal muscle. Measuring blood lactate concentration provides information regarding changes in glycolysis and the capacity of the anaerobic work [92]. Saliva lactate is possibly formed by passive diffusion from salivary glands and blood [64]. Blood lactate and saliva lactate are highly correlated, with most kinds of exercise [63]. It has been suggested that salivary lactate increases due to an increase in the lactate concentration of the blood, which leads to an increase in the permeability of the blood–saliva barrier during exercise [93]. Salivary concentrations of lactate during training have been estimated as lower than those of the lactate of the blood [62]. Lactate levels can also be used to assess the possibility of overtraining, as they decrease during intense exercise [60]. Lactate levels seem to be independent of individuals’ fitness and alteration in anxiety [61]. 

### 3.4. Oral Peroxides–Nitric Oxide 

One of the highest value components of the saliva antioxidant system is the enzyme nitric oxide [94]. The paramount importance of the salivary antioxidant system is to decompose hydrogen peroxide produced by bacteria. Then, the enzymes inactivate bacterial glycolytic enzymes, thus destroying the oral bacteria [95]. Exercise with moderate intensity increases the activity of salivary peroxidase. The lower the training power, the longer the peroxidase remains at the high activity level [72,96]. Exercise-induced stress also induces the production of salivary nitric oxide [97].

Oral peroxide increased only in beginner athletes and not in well-trained athletes and professionals [71,98]. So, it can be a measure of the adaptation of the subject to intense and heavy exercise. Furthermore, exercise increases saliva uric acid and total antioxidant activity, while the saliva lipid hydroperoxides decrease. Thus, it seems increment in uric acid and total antioxidant activity prevent the lipid hydroperoxides from being generated, making oral peroxide a marker of oxidative stress in saliva [70].

### 3.5. Salivary A-Amylase (sAA)

Some non-immunological salivary proteins can inhibit bacterial adherence to the oral cavity. One protein is a-amylase, which can bind to several oral bacteria [99,100,101]. Salivary a-amylase is the predominant enzyme in saliva. It is responsible for the degradation of starch and glycogen to maltose and has been used as a sympathetic nervous system activation biomarker [102]. Both a-amylase and cortisol of the saliva serve as markers to stress response of exertion [18]. However, salivary a-amylase activity is a more sensitive, exercise-induced stress marker than cortisol, as it is produced locally in the salivary glands, controlled by the autonomous nervous system. The cortisol is transported from blood to saliva [103]. Beta-adrenergic agonists are capable of stimulating salivary a-amylase release without increasing salivary flow [104].

Salivary a-amylase increased in acute exercise and the magnitude depended on exercise intensity [16,105]. Two hours of moderate exercise seems to lead to enhanced a-amylase activity [15]. Salivary a-amylase concentrations predict plasma catecholamine levels, particularly norepinephrine, under various stressful conditions and maybe a more direct endpoint of catecholamine activity [20]. Salivary a-amylase responses are quick within one to a few minutes, even faster from blood cortisol levels and it declines rapidly after removing the stress factor [20,65].

### 3.6. Salivary Cortisol (S-Cortisol)

Cortisol is the primary glucocorticoid produced by the adrenal cortex that regulates blood glucose homeostasis [65,106]. It is released in stressful situations and leads to an increase in blood-glycose [107]. High salivary cortisol concentrations are related to impaired insulin sensitivity [108]. Free cortisol is more increased than salivary cortisol [109]. After intensive training, periods with elevated cortisol associated with the mild hypoglycemic state seem to produce an immunosuppressive state and decrease plasma glutamine concentration [110]. Cortisol is responsible for 95% of the glucocorticoid activity in the human body [111]. The secretion of cortisol due to exercise is not immediate [37].

Salivary cortisol is expected to be decreased during non-exercising [112]. Low-intensity exercise also reduces the levels of salivary cortisol [112,113]. During moderate-intensity exercise, its levels remain almost stable [114,115]. Exercise of high intensity influences the secretory process of the adrenal cortex and starts cortisol releasing in adults and adolescents [30,116]. Heavy training significantly increases the amount of salivary cortisol immediately after exercise. Endurance exercise produces higher plasma cortisol than acute high-intensity exercise [79,117]. A study suggests that salivary cortisol is lower during water than land exercises [36]. This contrasts with another research work that indicates that the salivary cortisol concentrations similarly significantly decreased with water exercise and land stretching [35]. Physical activity seems to increase the diminished due to poor sleep quality awakening cortisol levels [110].

Physical fitness is associated with cortisol secretion during psychological stress [25,34,118,119,120]. Psychological stress factors can contribute to higher values of cortisol [34,118]. A relation between salivary cortisol and anxiety has been suggested [119,120]. Depressive patients before and 10 min after the exercise sessions appear to have significantly decreased levels of salivary-free cortisol [23,24,28]. In addition, physical exercise has been found to decline the rate of cortisol [12,22]. Salivary cortisol measurements can detect the circadian rhythm of the athletes, assisting in the prevention of overtraining syndrome [121].

Carbohydrates during exercise decrease glutamine depletion, cortisol and so the immune activity [122,123]. On the other hand, a diet with low carbohydrates suppresses immune activity and increases cortisol in plasma [123]. In addition, carbohydrate intake during prolonged exercise decreases stress hormones responses [123]. According to other studies, carbohydrates did not affect saliva flow rate and s-IgA concentration during a single bout of exercise [48,51]. However, post-exercise consumption of chocolate milk, which contains carbohydrates, proteins, fluid and electrolytes, is associated with lower saliva-cortisol response and higher saliva flow rate than water [124,125].

### 3.7. Steroids–Testosterone

Steroid hormones detectable in saliva include cortisol, androgens including testosterone and dehydroepiandrosterone (DHEA), estrogens and progesterone and aldosterone. Some serum components can transfer freely through the lipid-rich cell membrane into the salivary gland acinar cells and diffuse into the saliva. However, this mechanism is applied only to some lipid-soluble components such as steroid hormones. Salivary steroids are suggested to provide a more sensitive marker of changes than plasma ones [33].^,^ Salivary testosterone (sal-T), in unison with cortisol (sal-C), has been used as a marker of anabolic status [26,38]. Adrenal glands secrete DHEA. A strong relationship between salivary and plasma DHEA has been reported [126]. It has also been suggested as an analog in salivary testosterone measurements to assess exercise response in females [79].

Salivary measures of testosterone are a reliable indicator of its plasma concentrations [127]. Both testosterone and cortisol can be increased at a significant rate with hypertrophic exercising [128]. Salivary testosterone is increased linearly during exercise and reaches its peak after the end of the training [32]. Salivary testosterone seems to be a valuable tool to assess the performance of the athletes and their readiness to train at a certain intensity level and assist with the designing of workouts for optimal gains [78]. This can be explained as testosterone contributes to neuromuscular performance and the muscles’ long-term development [129]. The measurement of steroids of saliva samples throughout a competitive event can provide meaningful data regarding exercise’s psychological and physical stress and highlight overtraining [21,38].

### 3.8. Salivary Immunoglobulin A (s-IgA)

Immunoglobulin A (IgA) is the pre-dominant immunoglobulin in the mucosal immune system [130]. IgA is produced by long-lived plasma B cells, which are influenced by T cell-generated cytokines [131]. It is found in the saliva, intestinal secretions, bronchoalveolar lavage fluid, urine and other mucosal fluids and it is also associated with resistance to specific infections [132].

Salivary immunoglobulin A (s-IgA) plays an essential role in immunity as the first line of defense against potential pathogens [133]. Older people who follow a daily moderate-intensity exercise program appear to have higher levels of S-IgA, than others of the same age who do not exercise. In addition, moderate to intense exercise can increase the secretion of salivary S-IgA in older adults to improve their immune function [52]. S-IgA also presented an increased post-exercise when combined with a high carbonated diet, suggesting that carbohydrates enhance the immune activity during exercise [47]. On the contrary, others indicated no effect of carbohydrate ingestion on saliva immunoglobulin concentrations or secretion rates [51]. Finally, a study demonstrated that a fed or fasted state 2 h before exercise does not influence resting s-IgA [31].

As far as the relationship between exercise and s-IgA, the majority of the studies conclude that s-IgA decreases after exercise [27,134], others report no change [19,46] and others show increased levels of s-IgA post-exercise [17,43,50]. S-IgA measurement seems to be a good way which shows the over-training [135,136]. The s-IgA may decrease over prolonged periods of intensive training in elite athletes. This reduction is attributed to neurohormonal factors related to physical and psychological stress during intensive daily exercise [49]. In addition, no significant association between changes in s-IgA levels and those in cortisol levels during exercise [49,56]. Low temperatures, such as in ski races, might depress the activity of secretion of s-IgA [57].

High training loads can decrease s-IgA and suppress immune function, as lower concentrations of salivary IgA or chronic salivary IgA deficiencies are associated with an increased frequency of upper respiratory tract infections (URTI) [36,137]. However, more studies are required to clarify the relationship between the components of the saliva and the incidence of URTI. The coaches can use this information to predict athletes’ immune function to help reduce the risk of upper respiratory tract infection [42]. S-IgA fluctuation is seemed to be mirrored by the secretion of salivary free light chains [41].

### 3.9. Immunoglobulin G (IgG) & Immunoglobulin M (IgM)

All salivary immunoglobulins contribute to mucosal immunity and defense against upper respiratory tract infections. However, only a few studies have evaluated s-IgM and s-IgG, under physical exercise. It seems that s-IgG levels remain unchanged during exercise, while s-IgM levels decrease and are restored within 24 h [105].

### 3.10. Insulin-like Growth Factor 1 (s-IGF-1)

According to a study in young female volleyball players, free IGF-1 in saliva levels was decreased in well-trained athletes, compared with sedentary groups [59]. In contrast, in another study, salivary IGF-1 was increased after exercise, while plasma IGF-1 was not [58]. Salivary IGF-1 can be more sensitive. Training is suggested to increase human growth hormone hGh secretion, which is regulated by the hypothalamus. The increase seems to be attributed to insulin-like growth factor I (IGF-I) [138].

### 3.11. Salivary MicroRNAs

Salivary microRNAs can reflect critical biological processes related to a trauma, such as hypoxia, neurogenesis, axon repair and cell death. MicroRNAs, expressed from the saliva, seem to be an accurate non-invasive alternative to diagnose a traumatic brain injury due to a concussion [68,69].

### 3.12. Melatonin

Melatonin is a hormone found naturally in the human body, regulating sleep-wake cycles. Physical exercise during the afternoon can decrease melatonin secretion compared to the morning exercise. A non-invasive evaluation of melatonin can be performed [67].

### 3.13. Uric Acid

The effect of exercise in the concentration of uric acid in saliva has to be further investigated, as the available studies come to opposite conclusions. Aerobic exercise, such as long-distance running, has a significant impact, increasing the concentration of uric acid [70]. On the other hand, it seems that explosive physical exercise, such as short sprints, does not significantly influence the concentration of uric acid in saliva [40,139].

## 4. Conclusions

A significant part of the scientific literature has investigated the relation of physical exercise with the physical and biological properties of saliva. The shift in the composition of the saliva, during and after a workout, reflects the benefits of exercise, its potential risks and the capability of the saliva to serve as a health indicator. Saliva analysis can be used as a non-invasive method to measure exercise-induced changes, adaptation in hormones, lactate accumulation and shift in immunological markers. The type and the frequency of training, the physical condition and the athletes’ general health can influence hormones, immunoglobulins and saliva enzymes. The correlation between saliva and physical exercise has to be further investigated, especially for the organic components of the saliva. The available knowledge has to be applied to benefit sports activities. Athletes and coaches must consider monitoring salivary hormones during training or competitions for consistency or assessing overall workouts. Intelligent, easy to access affordable, non-invasive devices have to be further developed to take advantage of saliva’s information.

## Figures and Tables

**Table 1:** Primary outcomes of the included studies, separately for each investigated parameter of the saliva.

Evaluated Parameter of Saliva	First Author/Reference Number	Publication Date	Populations (Mean Age)	Primary Outcomes
A-amylase (sAA)	Yasuda N [15]	2021	11 Males cycling	Increase in A-amylase activity after moderately long-lasting exercise, regardless of exogenous carbohydrate availability
	Wunsch K [16]	2019	42 Males Acute exercise (24.1 ± 3) 42 Males placebo exercise (23.8 ± 2.3)	Increase in A-amylase concentration after moderate-to-high ergometer cycling. No difference in A-amylase peak level between habitual and acute exercise.
	Allgrove J [17]	2008	10 Males (23)	Increase A-amylase concentration after exercise, followed by a return to pre-existing values 1 h post-exercise. (Cycling) An increase in s-IgA was independent of exercise intensity.
	Li TL [18]	2004	8 Males (28.9 ± 1.8)	Increase in A-amylase activity after exercise (cycling, 60% VO max, 2 h)
	Walsh NP [19]	1999	8 Well trained males (25 ± 1)	Decrease in A-amylase concentration immediately after exercise. (cycling)
	Chatterton R [20]	1996	47 Medical students	Increase in A-amylase concentration. After exercise
Cortisol (S-cortisol)	Hough J [21]	2021	23 Active males, cycle ergometer (21 ± 3)	Increase in the s-cortisol immediately after the exercise. Decrease in the s-cortisol 30 min post-exercise
	Ushiki K [22]	2020	54 Participants (22)	Different rates of change in s-cortisol, depending on the intensity of the exercise. Decrease of s-cortisol levels after morning exercise. Increase of s-cortisol levels after afternoon exercise.
	Pearlmutter P [23]	2020	22 Athletes 26 Non-athletes (22 ± 3)	Decrease in s-cortisol concentration after exercise. Workout intensity affected changes among athletes and non-athletes. In non-athletes, the s-cortisol concentration decreased more compared to athletes.
	Rahman MS [24]	2019	945 Participants	Reduced s-cortisol levels after 12 weeks of physical exercise
	Wunsch K [16]	2019	42 Males Acute exercise (24.1 ± 3) 42 Males placebo exercise (23.8 ± 2.3)	Increase in s-cortisol concentration after moderate-to-high ergometer cycling. Habitual exercise can reduce s-cortisol peak concentrations.
	Wood CJ [25]	2018	164 Females (20 ± 3.4)	Increase in s-cortisol levels until 10 min of walking. The decline is s-cortisol levels after 10 min of walking. Participants with a more excellent fitness presented lower s-cortisol levels during walking,
	Crewther BT [26]	2013	14 Rugby players (23.3 ± 3.5)	Decrease in salivary cortisol before games.
	Gillum T [27]	2013	14 Marathon runners (43.7 ± 9.9)	Increase in s-cortisol concentration after exercise.
	Ida I [28]	2013	18 Outpatients (45.9 ± 13.3)	Decrease in s-cortisol before, immediately after and ten minutes after the exercise session.
	He CS [29]	2010	8 Basketball players (20.5 ± 0.3)	Increase in s-cortisol concentration during the intensive training and competition period
	Budde S [30]	2010	40 High school students (14.35)	Increase in s-cortisol post-exercise. No effect of physical activity level on the s-cortisol before and after exercise. (Sprinting)
	Allgrove J [31]	2009	16 Active adults (22 ± 4)	Increase in s-cortisol levels post-exercise.
	Thomas NE [32]	2009	17 School children (15.5 ± 0.4)	Increase in salivary cortisol after exercise. (Cycling)
	Allgrove J [17]	2008	10 Males (23)	No change in s-cortisol levels immediately after exercise. (Cycling) Increase in s-cortisol levels, 1 h after the training. Cortisol levels were higher in high-intensity training.
	Gozansky WS [33]	2005	12 Participants (23–65)	Significant correlation of salivary cortisol with serum cortisol.
	Neary JP [34]	2002	8 Physical education students Females (21 ± 2)	Significant correlation among the levels of s-cortisol, serum cortisol and urinary cortisol.
	Sugano A [35]	2000	7 Participants (61.9 ± 11.8)	Decrease in s-cortisol levels post-exercise. (Water exercise) Decrease in s-cortisol levels post-exercise. (Land stretching) No difference in s-cortisol levels among water and land physical exercise.
	Filaire E [36]	1996	10 Swimmers (18.5 ± 1.2) 14 Handball players 7 Sedentary participants	Increase in s-cortisol post-exercise only in handball players. Higher s-cortisol concentrations the following to exercise morning for handball players compared to swimmers and sedentary participants.
	Port K [37]	1991	6 Males	Increase in s-cortisol levels, especially in intensive training (Ergometer cycling)
	Cook NJ [38]	1986	8 Marathon runners (35.1 ± 8.1)	Increase in salivary cortisol during the marathon. Return to baseline levels, some hours after the marathon.
Cystatins	Sant’Anna M [39]	2019	20 Pentathletes (28 ± 6)	Increase in the secretion of S-type cystatins and cystatin C after aerobic and anaerobic exercise
Ferritin	Franco-Martinez L [40]	2019	18 Males (21.2 ± 4.2)	No difference in ferritin concentration in saliva after exercise. No correlation between blood lactate and salivary ferritin
Immunoglobulin A (s-IgA)	Yasuda N [15]	2021	11 Males	No change in s-IgA after moderately long-lasting exercise (2 h, cycling)
	Rapson A [41]	2020	46 Participants (23.7 ± 3.5)	Salivary free light chains (FLCs) follow the same pattern with the fluctuation of s-IgA.
	Tiernan C [42]	2020	19 Rugby players (19.7 ± 1.1)	A decrease in 65% or more of sIgA was associated with an increased risk within the following 2 weeks for contracting an upper respiratory tract infection. Not a significant association between s-IgA levels and training load.
	Engels HJ [43]	2018	50 Female participants	Increase in s-IgA concentration immediately after exercise. Return to the s-IgA pre-exercise levels 2 h after exercise.
	Gillum T [44]	2014	18 Participants	Increase in s-IgA, 1 h after exercise
	Gillum T [27]	2013	14 Marathon runners (43.7 ± 9.9)	Decrease in s-IgA concentration after exercise.
	He CS [29]	2010	8 Basketball players (20.5 ± 0.3)	Decrease in s-IgA concentration during the training and competition period
	Davison G [45]	2009	12 Males cycling	Increase in s-IgA after exercise
	Allgrove J [31]	2009	16 Active adults (22 ± 4)	Increase in s-IgA concentration post-exercise.
	Allgrove J [17]	2008	10 Males (23)	Increase in s-IgA concentration after exercise, followed by a return to pre-existing values 1 h post-exercise. (Cycling) An increase in s-IgA was independent of exercise intensity
	Sari-Sarraf V [46]	2006	8 Participants (24.1 ± 3.3)	No difference in s-IgA concentration and secretion rate during or after training. (standing, walking, jogging, cruising and sprinting)
	Costa RJS [47]	2005	32 Male triathletes, (32.1 ± 9)	Increased s-IgA concentration post-exercise in high carbohydrate consuming group.
	Tzai-Li Li [48]	2005	25 Participants (29)	Increase in s-IgA concentration after exercise, followed by a return to pre-existing values 2 h post-exercise. (Cycling)
	Tiollier E [49]	2005	21 Military cadets	No difference in s-IgA levels after 3-weeks of training. Increased s-IgA levels after 5 days of training and return to pre-training levels after 1 week of recovery.
	Li TL [18]	2004	8 Males cycling (28.9 ± 1.8)	Increase in s-IgA concentration after exercise (60% VOmax, 2 h) No difference in s-IgA secretion rate after training.
	Akimoto T [50]	2003	45 Participants (64.9 ± 8.4)	Increase in s-IgA concentration 12 months of physical exercise training. Increase in s-IgA concentration after 4 months of physical exercise training.
	Nehlsen-Cannarella S [51]	2000	20 Elite female rowers (22.6 ± 0.5) 19 Non-athletic females (24.6 ± 0.8)	77% Higher s-IgA concentration in the rowers compared to non-athletes
	Walsh NP [19]	1999	8 Well trained males (25 ± 1)	S-IgA secretion rate was not affected by the exercise. (cycling)
	Shimizu K [52]	2007	114 Men (71.6 ± 0.4) 170 Women (71 ± 0.3) Elderly volunteers	The S-IgA flow rate and secretion rate increased when physical activity was improved.
	Gleeson M [53]	1995	26 Elite swimmers (16–24) 12 Athletic participants (19–41)	Increase in s-IgA levels in professional swimmers immediately after exercise. There is no difference in pre-exercise s-IgA levels in professional swimmers compared to the athletic participant.
	Mackinnon LT [54]	1994	10 Joggers (20–35) 7 Marathon runners (20–35) 14 Swimmers (16–20)	No change in s-IgA secretion rates in Joggers after exercise, irrespective of exercise intensity. Decrease in s-IgA secretion rates in marathon runners after 90 min of running after the first day of exercise. There is no difference in s-Iga concentration, in stale compared to well-trained swimmers, during a season of 6 months.
	Mackinnon LT [55]	1993	12 Physical education students (17–25)	Increase in S-IgA concentration after training, A decline in S-IgA flow rate after training. (Cycling)
	McDowell SL [56]	1992	24 Novice runners (22.1 ± 3)	Decrease in s-IgA levels immediately after the exercise. Increased compared to prior exercise, s-IgA concentration 1 h post-exercise. S-IgA levels are independent of salivary cortisol.
	Tomasi T [57]	1982	8 Nationally ranked skiers (23.5) 8 Non-competitive skiers	Decrease in s-IgA levels after exercise. (skiing) Lower s-IgA levels of the skiers compared to non-competitive athletes.
Immunoglobulin G (s-IgG)	Nehlsen-Cannarella S [51]	2000	20 Elite female rowers (22.6 ± 0.5) 19 Non-athletic females (24.6 ± 0.8)	No difference in s-IgG concentration among the rowers compared to non-athletes
	Gleeson M [53]	1995	26 Elite swimmers (16–24) 12 Athletic participants (19–41)	Higher s-IgG levels in professional swimmers compared to the athletes post-exercise. There is no difference in s-IgG levels among the professional swimmer and athletic participants post-exercise.
	Mackinnon LT [55]	1993	12 Physical education students (17–25)	Increase in S-IgG concentration after training, A decline in S-IgG flow rate after training. (Cycling)
	Tomasi T [57]	1982	8 Nationally ranked skiers (23.5) 8 Non-competitive skiers (25.5)	Same s-IgG levels prior and post-exercise. No difference in s-IgG levels among the skiers and non-competitive athletes.
Immunoglobulin M (s-IgM)	Nehlsen-Cannarella S [51]	2000	20 Elite female rowers (22.6 ± 0.5) 19 Non-athletic females (24.6 ± 0.8)	No difference in s-IgM concentration among the rowers compared to non-athletes
	Gleeson M [53]	1995	26 Elite swimmers (16–24) 12 Athletic participants (19–41)	Higher s-IgM levels in professional swimmers compared to the athletes post-exercise. There is no difference in s-IgM levels among the professional swimmer and athletic participants post-exercise.
	Mackinnon LT [55]	1993	12 Physical education students (17–25)	Increase in S-IgM concentration after training, A decline in S-IgM flow rate after training. (Cycling)
Insulin-like growth factor I (s-IGF-I)	Antonelli G [58]	2009	18 Cyclists (19 ± 1)	Increase in s-IGF-I after exercise.
	Antonelli G [59]	2007	15 Volleyball players 14 Sedentary females	Lower s-IGF-I in athletes compared to sedentary females before exercise.
Lactate	Almasi G [60]	2021	31 Elite adolescent athletes	Increase in the concentration of salivary lactate after exercise. (200 m freestyle swimming)
	Hermann R [61]	2019	32 Males (24.3 ± 3.3)	Increase in the concentration of salivary lactate after ergometer
	Franco-Martinez L [40]	2019	18 Males (21.2 ± 4.2)	Increase in the concentration of lactate in the saliva after exercise. (Sprinting) Lactate in saliva was correlated with blood lactate only in untrained subjects
	Santos RV [62]	2006	15 Expert marathon racers	Increase in the concentration of salivary lactate after 18 km of running.
	Segura R [63]	1996	9 Amateur sportsmen (22.2 ± 1.9)	Increase in the concentration of salivary lactate both for anaerobic and aerobic exercise.
	Ohkuwa T [64]	1995	7 Long-distance runners (18.6 ± 0.8) 5 Sprinters (19.3 ± 1.1)	Increase the salivary lactate concentration both in 400-m and in the 3000-m run. Higher lactate concentration after 400-m run for sprinters compared to long-distance runners.
	Port K [37]	1991	6 Males	Steadily increase of the lactate throughout the exercise
Lysozyme, lactoferrin	Gillum T [44]	2017	11 Participants (20.3 ± 0.8)	Increase in lysozyme secretion rate after exercise. (ran for 45 min at 75% of VO2peak)
	De Feo P [65]	1989	9 Male Participants (21.1 ± 1.1) 9 Female (22.4 ± 2.4)	Increase in lactoferrin secretion rate after exercise. Increase in lysozyme secretion rate after exercise.
	Gillum T [27]	2013	14 Marathon runners (43.7 ± 9.9)	Increase in lactoferrin concentration after exercise. Lysozyme concentration does not change during exercise.
	He CS [29]	2010	8 Basketball players (20.5 ± 0.3)	Decrease in lactoferrin concentration during the training and competition period
	West NP [66]	2010	17 Elite rowers (24.3 ± 4) 18 Sedentary individuals (27.2 ± 7)	60% decreased lactoferrin concentration before exercise in elite rowers compared to sedentary individuals 50% increase in the lysozyme concentration and 50% increase in lactoferrin after graded exercise.
	Allgrove J [17]	2008	10 Males (23)	Increased lysozyme concentration after exercise for 1 h. (Cycling)
Melatonin	Carlson LA [67]	2019	12 Regularly exercising men	Increased salivary melatonin after morning exercise compared to afternoon exercise.
MicroRNAs	Hicks S [68]	2020	Former football players (73 ± 8) Younger participants (20 ± 5)	Non-invasive measurement of saliva miRNAs, (miR-340-5p, miR-339-3p, miR-361-5p, miR-28-3p) may have utility to identify individuals at risk for chronic concussion symptoms.
Nitric Oxide	Di Pietro V [69]	2018	52 Rugby Athletes (26)	Differentially expressed miRNAs could be particularly suitable for concussion assessment.
	Gonzalez D [70]	2008	24 Participants (27.2 ± 9.6)	No change in nitric concentration after aerobic exercise.
	Panossian AG [71]	1999	109 Athletes (32–44)	Increase in nitric oxide concentration after exercise in amateur athletes.
Peroxides	Damirchi A [72]	2010	10 University students	Increase in peroxide secretion rate at the 75%VO(2 max) after exercise. Decrease 1 h after exercise.
	Gillum T [27]	2013	14 Marathon runners (43.7 ± 9.9)	Salivary flow rate not changed by the exercise.
	Damirchi A [72]	2010	10 University students	The salivary flow rate does not change by the exercise. (Treadmill runs)
	Allgrove J [31]	2009	16 Active adults (22 ± 4)	Decrease in saliva flow rate during exercise, followed by a return to pre-existing values 1 h post-exercise. (cycling)
	Allgrove J [17]	2008	10 Males (23)	Salivary flow rate not changed by the exercise. (Cycling)
	Shimizu K [52]	2007	114 Men (71.6 ± 0.4) 170 Women (71 ± 0.3) Elderly volunteers	No difference in salivary flow rate when physical activity is improved.
	Sari-Sarraf V [46]	2006	8 Participants (24.1 ± 3.3)	Decrease in saliva flow rate during exercise. (standing, walking, jogging, cruising and sprinting) Duration of exercise significantly influenced the reduction in saliva flow rate.
	Tzai-Li Li [48]	2005	25 Participants (29)	Decrease in saliva flow rate during exercise, followed by a return to pre-existing values 1 h post-exercise. (cycling)
	Li TL [18]	2004	8 Men cycling (28.9 ± 1.8)	Decrease in saliva flow rate after exercise (60% VOmax, 2 h)
	Akimoto T [50]	2003	45 Participants (64.9 ± 8.4)	No difference in saliva flow rate after 12 months of physical exercise training.
	Walsh NP [73]	2002	15 Cyclists	Decrease in saliva flow rate after exercise
	Nehlsen-Cannarella S [51]	2000	20 Elite female rowers (22.6 ± 0.5) 19 Non-athletic females (24.6 ± 0.8)	No difference in saliva secretion rate among the professionals and non-athletic participants.
	Walsh NP [19]	1999	8 Well trained males (25 ± 1)	The saliva flow rate was not affected by the exercise. (cycling)
	Blannin A [74]	1998	18 Male with mixed physical fitness (23 ± 1)	Saliva flow rate reduced by moderate or high-intensity exercise
	Steerenberg P [75]	1997	42 Triathletes (34.1 ± 7.3)	Reduced saliva flow rate after the race
	Pilardeau P [76]	1990	12 Male	In normoxia or hypoxia, there is no difference in saliva flow rate after exercise. However, in the situation of acute hypoxia, reduced saliva flow rate after exercise.
Testosterone	Hough J [21]	2021	23 Active males, cycle ergometer (21 ± 3)	Increase in the salivary testosterone immediately after the exercise. Decrease in the salivary testosterone 30 min post-exercise
	Cook CJ [77]	2014	20 Rugby players (21.5 ± 1.4)	Increase in salivary testosterone after functional improvement. (Training)
	Crewther BT [26]	2013	14 Rugby players (23.3 ± 3.5)	Increase in salivary testosterone before winning games.
	Budde S [30]	2010	40 High school students (14.35)	Increase in the salivary testosterone after exercise. No effect of activity level on the salivary testosterone prior and after exercise. (Sprinting)
	Crewther BT [78]	2010	4 Male (20.8 ±3.5) 4 Female (20.8 ± 4.6) Olympic weightlifters	Significant correlation of pre-workout salivary testosterone, with the Olympic total lift, only for male weightlifters.
	Thomas NE [32]	2009	17 School children (15.5 ± 0.4)	Increase in salivary testosterone after exercise. (Cycling)
	Filaire E [79]	2000	14 National handball players (24.1 ± 2.6) 10 Sedentary women (23.5 ± 3.4)	Higher salivary testosterone for sedentary women, compared to professional players, at resting. Correlation among testosterone and dehydroepiandrosterone (DHEA).
	Cook NJ [38]	1986	8 Marathon runners (35.1 ± 8.1)	Increase in salivary testosterone during the marathon. Increased salivary testosterone concentration, the days after the marathon.
Uric acid	Franco-Martinez L [40]	2019	18 Males (21.2 ± 4.2)	No difference in uric concentration in saliva after exercise. Significantly negative correlation between uric acid and blood lactate.
	Gonzalez D [70]	2008	24 Participants (27.2 ± 9.6)	Increase in uric acid by aerobic exercise.
